# Acute urinary retention and urinary tract infection after short‐course urinary drainage in colon or high rectum anastomoses: Post hoc analysis of a multicentre prospective database from the GRACE group

**DOI:** 10.1111/codi.16184

**Published:** 2022-05-31

**Authors:** Aurélien Venara, Jean François Hamel, Jean‐Marc Régimbeau, Julien Gillet, Jean Joris, Eddy Cotte, Karem Slim, the GRACE group, Orabi Nora Abbes, Elodie Agut, Pascal Alfonsi, Asma Alili, Jibba Amraoui, Adeline Andre, Jean‐Marc Arimon, Laurent Arnalsteen, Robert Asztalos, Cyril Audouy, Ophélie Aumont, Sylvain Auvray, Hubert Baietto, Gregorio Balbo, Maryse Barreau Aguilera, Nathan Beaupel, Nathan Beaupel, Zeineb Ben Lazreg, Sophie Beguinot‐Holtzscherer, Jean‐Paul Beller, Arnauld Bellouard, Imed Ben Henda, Mohamed Bentamene, Pierre Bernard, Nicolas Berthon, Amélie Biblocque, Thomas Bievre, Marco Bilosi, Benjamin Blanc, Sébastien Blaye‐Felice, Adrien Blatt, Damien Blehaut, Anne Bock, Jean‐Pierre Bongiovanni, Marc Bonnet, Noredinne Bouarroudj, David Boissier, Henry Boret, Ruddy Borg, Zoheir Bouchair, François Bouchard, Mohamed Boumadani, David Bounicaud, Olivier Bourdeix, J. C. Bourseau, Guillaume Bozio, Dorothée Brachet, Amine Brek, Nicolas Briez, Carole Buisset‐Subiran, Brigitte Calvet, Anna Cartaux‐Taieb, Marie Castiglioni, Maryline Catinois, Mael Chalret du Rieu, Claire Chalumeau, Gerald Chambrier, Reza Chamlou, Nathalie Chapel, Pierre Chenet, Pierre Chirac, Seddik Chokkairi, Xavier Chopin, Niki Christou, Etienne Chuffart, François Corfiotti, Carmen Craus, Emmanuel Cuellar, Gilles Dardenne, Nicola de Angelis, Ugo de Ioro, Fabien Dechanet, Rachel Dellis, Laurence Demasles, Christine Denet, Benjamin Deroo, Véronique Desfourneaux‐Denis, Sylvain Dileon, Richard Douard, Carlos Dorado, Eva Dorscheid, Frédéric Dumont, François Durame, Emilie Duchalais, Aurélien Dupre, Sophie Dufraisse, Mohamed Amine Elghali, Emmeric Hutin, Aloui Emna, Eric Essome, Nathalie Fabre, Virginie Faivre, Jean‐Luc Faucheron, Patrick Favoulet, Philippe Fernou, Olivier Firtion, Renaud Flamein, Sabina Florea, Christophe de la Fontaine, Damien Forestier, Erwann Fourn, Dacian Vasile Frentiu, Romain Frisoni, Alain Frisoni, Thomas Gautier, Florent Genty, Sebastian Georgeanu, Adeline Germain, Stéphanie Gibert, Bruno Gilbert, Benoit Gignoux, Nicolas Goasguen, Pierre Goubault, Philippe Gres, Jérémie Guedj, Bruno Guignard, Jean Gugenheim, Caroline Guaquiere, Jean Luc Guiot, David Guinier, Kamel Hail, Caroline Hatwel, Elean Iatan, Thérèse Janecki, Thomas Jany, Jerome Jaspart, Frédéric Journe, Lionel Jouffret, Aicha Kassoul, Fehmi Kattou, Philipe Keller, Thomas Knepfler, Tarik Khouri, Konstantinos Kothonidis, Pierre Landreau, Guillaume Langlois, Gerald Le Bartz, Sébastien Lebas, Daniel Leonard, David Leonard, Julien Leporrier, Guy Lescure, Romain Lewandowski, Antonella Liddo, Jean‐Hugues Longeville, Ioan Lucescu, Antoine Mariani, Pascale Mariani, Gwenaelle Martin, Olivier Martinet, Damien Massalou, Jean‐Loup Massard, François Mauvais, Davide Mazza, Jean Mbuyamba Katapile, Fabrice Milou, Frantz Mirre, Caroline Mor Martinez, Alexandre Mensier, Claude Mergui, Jean‐Philippe Mestrallet, Caroline Meyer, Nicolas Mocellin, Serge Montagne, Omar Naseef, Marion Orville, Sandrine Ostermann‐Bucher, Mehdi Ouaissi, Xavier Paqueron, Cyrielle Paquet, Laurent Passebois, Virginie Pichot‐Delahaye, Marc Pillet, Jean Charles Pottie, Laurent Plard, Fleur Plumereau, James Poincenot, Marie Poisblanc, Benoit Poupard, Jan Martin Proske, Pierre Puche, Olivier Raspado, Romain Riboud, Barivola Rakotoarisoa, Kevin Raynaud, Thierry Razafindratsira, Myriam Renaud, Didier Rio, Didier Rio, Jeremie Ripoche, Benjamin Roussel, Marc Saint Denis, Pascale Salaun, Pierre Yves Sage, Marie‐Lorraine Scherrer, Franck Sirisier, Boudewijn Smeets, Milan Smejkal, Jean‐Philppe Steinmetz, Marion Tavernier, Remy Thievenaz, Mihaela Tirca, Laurence Toque, Elhocine Triki, Dimitri Tzanis, Bernard Vacher, Serge Vanwymeersch, Estelle Vauclair, Romain Verhaeghe, Victoria Vetrila, Christine Vieuille, François Vermeulen, Jean‐Charles Vignal, Christian Voilin, Pierre de Wailli, Albert Wolthuis, Sophie Zaepfel

**Affiliations:** ^1^ Department of Visceral and Endocrinal Surgery University Hospital of Angers Angers Cedex 9 France; ^2^ Faculty of Health Department of Medicine Angers France; ^3^ Univ Angers, [CHU Angers], HIFIH, SFR ICAT, F‐49000 Angers, France University of Angers Angers France; ^4^ Department of Biostatistics, Maison de la Recherche University Hospital of Angers Angers Cedex 9 France; ^5^ Service de Chirurgie Digestive CHU Amiens Picardie et Université de Picardie Jules Verne Amiens France; ^6^ Unité de Recherche Clinique SSPC (Simplifications des Soins des Patients Complexes) UR UPJV 7518 Université de Picardie Jules Verne Amiens France; ^7^ Department of Anaesthesiology CHU Liège Liège Belgium; ^8^ Department of Visceral Surgery, Centre Hospitalier Lyon‐Sud CHU Lyon Pierre‐Bénite Cedex France; ^9^ Université de Lyon Lyon France; ^10^ Department of Visceral Surgery CHU Clermont‐Ferrand Clermont‐Ferrand France

**Keywords:** acute urinary retention, colorectal surgery, enhanced recovery programme, urinary drainage, urinary tract infection

## Abstract

**Aim:**

The aim was to define the risk factors for acute urinary retention (AUR) and urinary tract infections (UTIs) in colon or high rectum anastomosis patients based on the absence of a urinary catheter (UC) or the early removal of the UC (<24 h).

**Method:**

This is a multicentre, international retrospective analysis of a prospective database including all patients undergoing colon or high rectum anastomoses. Patients were part of the enhanced recovery programme audit, developed by the Francophone Group for Enhanced Recovery after Surgery, and were included if no UC was inserted or if a UC was inserted for <24 h.

**Results:**

In all, 9389 patients had colon or high rectum anastomoses using laparoscopy, open surgery or robotic surgery. Among these patients, 4048 were excluded because the UC was left in place >24 h (43.1%) and 97 were excluded because the management of UC was unknown (1%). Among the 5244 colon or high rectum anastomoses patients included, AUR occurred in 5.2% and UTI occurred in 0.7%. UCs were in place for <24 h in 2765 patients (52.7%) and 2479 did not have UCs in place (47.3%). Multivariate analysis showed that management of the UC was not significantly associated with the occurrence of AUR and that risk factors for AUR were male gender, ≥65 years old, having an American Society of Anesthesiologists score ≥3 and receiving epidural analgesia. Conversely, being of male gender was a protective factor of UTI, while being ≥65 years old, having open surgery and receiving epidural analgesia were risk factors for UTIs. The management of the UC was not significantly associated with the occurrence of UTIs but the occurrence of AUR was a more significant risk factor for UTIs.

**Conclusion:**

UCs in place for <24 h did not reduce the occurrence of AUR or UTI compared to the absence of UCs.

**What does this paper add to the literature?**

There is no consensus on the gold standard in urinary catheter (UC) management after colon or high rectum anastomoses. This study shows that a UC is not mandatory, since a UC left in place ≤24 h does not reduce the risk of acute urinary retention, nor does it increase the risk of urinary tract infection.

## INTRODUCTION

Recent American guidelines recommend that, after colon or high rectum anastomoses, a urinary catheter (UC) should be removed 24 h following the procedure [1]. However, current practice varies widely and some studies point to the feasibility of UC abstention in colon surgery in the context of enhanced recovery programmes (ERPs) [2, 3].

UCs are placed in order to reduce the risk of acute urinary retention (AUR) after surgery. However, they could also increase the risk of urinary tract infection (UTI). AUR is reported as a complication in 4%–22% of cases [4], while UTIs are reported in 1.4%‐4% of cases [2].

Traditionally, colon or high rectum anastomosis patients had UCs in place for up to 3 days after surgery, but ERPs now make it possible for patients to be fitted with fewer surgical drains [5]. Several studies have shown that short‐term placement of UCs does not increase the risk of AUR, whilst it does reduce the risk of UTIs in colon or high rectum anastomosis patients [2].

With only a few studies available in the literature, there is currently little evidence in favour of the abstention of UCs during colon surgery. To the best of our knowledge, only two studies exist that consider the feasibility of this type of management. One of these is a small French cohort study, which reported that laparoscopic colonic resection without a UC is feasible and is associated with a 9% risk of AUR and a 1.5% risk of UTI [3]. In this study, no comparison was made with a control group. In another recent cohort study, the authors assessed the rates of AUR in patients both with and without UCs in place during colon or rectal surgery [2]. Deferral or early removal of UCs was feasible and safe without an increased risk of AUR, but the rate of patients with no UC in place was <5% [2].

There is therefore a need for strong evidence in order to publish recommendations on UC management in colon or high rectum anastomosis.

The aim of this study was to define the risk factors of AUR and UTI in colon or high rectum anastomoses patients based on the short‐course management of UCs (no UC or UC removed within 24 h).

## MATERIALS AND METHODS

### Study design

All patients undergoing colon and high rectum anastomoses who had been included in the ERP audit developed by the Francophone Group for Enhanced Recovery after Surgery (GRACE) between 1 December 2012 and 31 May 2021 were included in a retrospective analysis. Patients came from 66 centres in France, Algeria, the Netherlands, Switzerland and Belgium.

**FIGURE 1:**
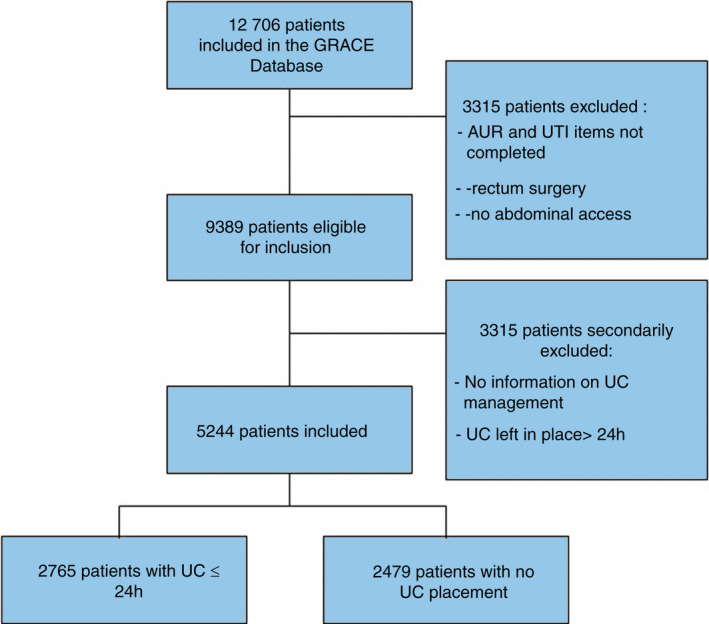
Flow chart of our population

Patients were included if they underwent any form of colon or high rectum anastomosis performed with open, laparoscopic or robotic surgery. Patients for whom the occurrence of AUR or UTI was not recorded or for whom the UC management was not recorded were excluded. Patients who had UCs left in for more than 24 h were later excluded (Figure 1). The decision regarding whether or not to leave the UC in position for more than 24 h was at the individual surgeon's discretion, and it was taken before the surgery as per the hospital's ERP.

Some of these patients were already included in a study assessing risk factors or mechanisms for postoperative ileus [6, 7, 8].

The database has been accredited to handle healthcare data (in accordance with the French Ministerial Decree of 4 January 2006). It was developed by GRACE and approved by the French National Data Protection Authority, registration number 2014#1817711. All patients were informed, and they agreed with the process.

### Groups and end‐points

The study aimed to assess the risk of AUR and UTI in colon and high rectum anastomosis with short‐term UC use. UC placement (early removal [≤24 h] or no placement) was left to the surgeon's discretion.

AUR was defined by the inability to ensure bladder voiding and the necessity of installing or replacing a UC during the hospital stay.

UTIs were suspected in cases of burning urination and they were confirmed by bacteriological analysis of urine samples during the hospital stay. Antibiotic therapy was recommended for treating affected patients.

### Collected data

All data were collected prospectively in order to make the audit recommended in all ERPs. Data collected included the following:

- *demographic characteristics:* gender, age, body mass index (BMI), American Society of Anesthesiologists (ASA) score, nature of the pathology (cancer vs. benign), neoadjuvant radiochemotherapy;
- *surgical characteristics:* surgical access (laparoscopy, robotic, open surgery), site of surgery (right colectomy, left colectomy [e.g., left colectomy, sigmoid colon resection, high rectum resection], other and Hartmann reversal), conversion to open surgery, duration of surgery;
- *perioperative management*, defined as the percentage of compliance with the ERP. The items used to check compliance with the ERP were preoperative concealing, preoperative premedication, reduced fasting, preoperative carbohydrates, mini‐invasive access, perioperative antibiotic prophylaxis, prevention of hypothermia, multimodal analgesia, prevention of nausea, avoiding unnecessary drainage, surgical drainage, perioperative injection of dexamethasone, adapted fluid loading, ablation of the infusion before postoperative day 3, postoperative multimodal analgesia, thromboprophylaxis, mobilization and feeding on the day of surgery;
- *use of epidural analgesia*.

### Statistical analysis

Continuous data were described as means and standard deviations, and they were compared to each other with the *t* test. Categorical data were described using percentages, and they were compared to each other using the chi‐squared test.

The relationship between the management of the UC and AUR or UTI was evaluated using logistic regression models that took gender, BMI, age, ASA score, history of cancer, surgical access, duration of the surgery, type of surgery, compliance with the ERP and epidural analgesia as possible cofounders. The model was validated by analysing the residual distributions. All the tests were two‐sided with type I error set at 0.05. The analyses were performed using Stata 14.2 software.

## RESULTS

### Description of the population

The database contained information on 12 706 patients. Of these, 322 patients were excluded due to a lack of details regarding the occurrence of AUR or UTI (2.5%). Another 2772 patients were excluded because low or mid‐rectal surgery was performed (21.8%) and 223 were excluded because they had elective surgeries that did not require laparoscopic or open surgery (1.7%). The remaining 9389 patients had undergone colon or high rectum anastomosis through either laparoscopy, open or robotic surgery (Figure 1). Among these patients, 97 were excluded because there was no information on the UC management (1%) and 4048 were excluded because the UC was left in place >24 h (43.1%). In all, the data of 5244 patients were analysed: 2765 patients had a UC installed for the short term (52.7%) and 2479 had no UC installed (47.3%).

Women made up 2671 (50.9%) of the patients included; 2735 patients were ≥65 years old (52.1%) and 863 had a BMI ≥ 30 kg/m^2^ (16.5%). AUR occurred in 275 patients (5.2%) and UTIs occurred in 37 patients (0.7%).

At baseline, the two groups differed in terms of gender, age, BMI, ASA score, type of surgery, need for conversion to open surgery, duration of the surgery and whether or not analgesia was given with an epidural (Table 1). The occurrence of AUR did not differ between the short‐term UC group (5.2%) and the no UC group (5.3%) (*p* = 0.80). Similarly, the occurrence of UTIs did not differ between these groups (0.9% vs. 0.5%, respectively; *p* = 0.052).

**TABLE 1:** Comparison of demographic, perioperative and postoperative characteristics of the group of patients with short‐course UC or no UC

	Short‐course UC, *n* = 2765 (52.7%)	No UC, *n* = 2479 (47.3%)	*p*
Gender female	1407 (50.9%)	1267 (51.1%)	0.87
BMI ≥30 kg/m^2^	464 (17%)	399 (16.3%)	0.47
Age ≥65 years	1380 (50%)	1355 (54.9%)	**0.0005**
ASA score			
1–2	2127 (76.9%)	1822 (73.5%)	**0.005**
3–4	638 (23.1%)	656 (26.5%)	
Cancer	1621 (58.6%)	1397 (56.3%)	0.09
Surgical access			
Laparoscopic access	2253 (81.8%)	2058 (83.1%)	0.32
Robotic access	57 (2.07%)	56 (2.26%)	
Open access	445 (16.1%)	364 (14.7%)	
Conversion	136 (4.9%)	91 (3.7%)	**0.03**
Duration of surgery (min)			
≤90	383 (14.4%)	549 (23.2%)	**<0.001**
90–180	1602 (60.1%)	1374 (58.1%)	
>180	680 (25.5%)	443 (18.7%)	
Type of colon surgery			
Right colectomy	1022 (39.5%)	1170 (48.6%)	**<0.001**
Left colectomy	1219 (47.1%)	795 (33.1%)	
Hartmann reversal	127 (4.9%)	282 (11.7%)	
Other	222 (8.6%)	159 (6.6%)	
Colon resection	2537 (91.7%)	2127 (85.8%)	**<0.001**
Percentage of compliance with ERP	84.1 ± 12.1	86.8 ± 9.4	**<0.001**
Epidural analgesia	283 (10.2%)	97 (3.9%)	**<0.001**
Urinary tract infection	26 (0.9%)	12 (0.5%)	**0.052**
AUR	143 (5.2%)	132 (5.3%)	0.80

Abbreviations: ASA, American Society of Anesthesiologists; AUR, acute urinary retention; BMI, body mass index; ERP, enhanced recovery programme; UC, urinary catheter.

Bold is for significant differences.

### Risk factors for AUR

Multivariate analysis demonstrated that being of male gender (OR = 1.45; 95% CI 1.2–2), ≥65 years old (OR = 1.52; 95% CI 1.1–2) and having an ASA score ≥3(OR = 1.49; 95% CI 1.1–2) were risk factors for AUR (Table 2). Epidural analgesia was a risk factor for AUR (OR = 1.83; 95% CI 1.2–2.8), while management of the UC was not significantly associated with the occurrence of AUR (OR = 1.17; 95% CI 0.9–1.5).

**TABLE 2:** Risk factors for acute urinary retention in colon or high rectum anastomoses

	Odds ratio	95% confidence interval	*p* > *z*
Gender male	1.549	1.185–2.024	0.001
BMI ≥30 kg/m^2^	0.792	0.550–1.142	0.212
Age ≥65 years	1.529	1.138–2.054	0.005
ASA score ≥3	1.487	1.114–1.986	0.007
Cancer	0.936	0.699–1.252	0.654
Surgical access (ref: laparoscopy)			
Robotic	0.764	0.237–2.468	0.653
Open access	1.253	0.880–1.784	0.210
Conversion in open surgery	0.845	0.436–1.639	0.618
Duration of surgery (ref: ≤90 min)			
90–180 min	1.181	0.797–1.751	0.408
≥180 min	1.440	0.919–2.256	0.112
Type of colon or high rectum anastomoses (ref: right colectomy)			
Left colectomy or high rectum resection	0.960	0.713–1.294	0.791
Hartmann reversal	0.404	0.121–1.347	0.140
Other	0.716	0.354–1.447 0.249–2.053	0.352
Colectomy (ref: no resection)	0.715		0.533
Compliance with ERP	0.989	0.977–1.001	0.075
Epidural analgesia	1.830	1.211–2.767	0.004
No urinary catheter (ref: early removal)	1.169	0.89–1.53	0.260

Abbreviations: ASA, American Society of Anesthesiologists; BMI, body mass index; ERP, enhanced recovery programme.

### Risk factors for UTIs

Multivariate analysis demonstrated that being ≥65 years old (OR = 2.84; 95% CI 1.2–6.6) was a risk factor for UTIs while being of male gender was a protective factor for this complication (OR = 0.49; 95% CI 0.2–0.9) (Table 3).

**TABLE 3:** Risk factors for urinary tract infection in colon or high rectum anastomoses

	Odds ratio	95% confidence interval	*p* > *z*
Gender male	0.486	0.243–0.972	0.041
BMI ≥30 kg/m^2^	1.392	0.636–3.047	0.407
Age ≥65 years	2.844	1.225–6.602	0.015
ASA score ≥3	2.015	0.987–4.112	0.054
Cancer	0.904	0.431–1.896	0.790
Surgical access (ref: laparoscopy)			
Robotic	2.245	0.288–17.481	0.440
Open access	2.231	1.001–4.971	0.050
Conversion in open surgery	2.368	0.684–8.191	0.174
Duration of surgery (ref: ≤90 min)			
90–180 min	1.275	0.452–3.597	0.646
≥180 min	1.221	0.376–3.967	0.740
Type of colon or high rectum anastomoses (ref: right colectomy)			
Left colectomy or high rectum resection	1.406	0.652–3.032	0.385
Hartmann reversal	1.731	0.102–29.262	0.704
Other	1.440	0.317–6.536	0.637
Colectomy (ref: no resection)	1.127	0.089–14.274	0.926
Compliance with ERP	1.018	0.983–1.053	0.315
Epidural analgesia	2.710	1.145–6.418	0.023
No urinary catheter (ref: early removal)	0.546	0.264–1.130	0.103
AUR	3.833	1.613–9.104	0.002

Abbreviations: ASA, American Society of Anesthesiologists; AUR, acute urinary retention; BMI, body mass index; ERP, enhanced recovery programme.

The management of UCs was not significantly associated with the occurrence of AUR (OR = 0.54; 95% CI 1.1–6.4), while open surgery (OR = 2.23; 95% CI 1–4.9) and epidural analgesia (OR = 2.71; 95% CI 1.1–6.4) were risk factors for UTIs.

The occurrence of AUR was the most significant risk factor of UTI (OR = 3.83; 95% CI 1.6–9.1).

## DISCUSSION

Despite recommendations to remove the UC early, 43% of patients in our cohort still had a UC in place for >24 h. This is significantly more than the 24%–30% of UCs left in place reported in the literature [2, 9]. We are unable to explain this difference; it highlights the importance of providing strong evidence in order to standardize practice and to manage the UC in the best manner possible, especially within the ERP setting. As this was a retrospective study based on a prospective database, we were unable to determine the reasons why the UC was left in place. Most of the patients kept the UC because it was part of that particular hospital's ERP, although some patients may have kept the UC as the surgeon felt that they may have been at risk of AUR. This led to a bias that should be taken in account when interpreting the results, as it could lead to an underestimation of the incidence of AUR in our population. Our study compared the risk factors for AUR and UTI, depending on whether or not a UC was installed for a short duration, less than 24 h.

The importance of limiting the time for which the UC is in place to less than 24 h has already been highlighted in the literature. A recent meta‐analysis reports that while this shorter period could increase the risk of needing to re‐catheterize a patient (relative risk 1.81, 95% CI 1.35–2.41), it would decrease the risk of symptomatic catheter‐associated UTIs (relative risk 0.52, 95% CI 0.45–0.61) [10]. In abdominal surgery, a randomized controlled study compared the rate of UTIs and AUR in patients with short‐ versus long‐course UCs. The rate of UTIs was higher in patients with long‐term UCs (14% vs. 2%), while the rate of AUR was similar [11]. Similarly, in a specific colorectal cohort of patients managed in an ERP context, early ablation of UC was not associated with AUR [12]. Thus, as the recommendations advocate early removal of UCs, we specifically focused our interest on patients who had a UC installed for a short period of time versus those who had no UC installed.

In the literature, AUR has been reported to occur in 4% and 22% of patients, which is similar to our cohort. The literature also reports higher number of UTIs than we found in our cohort (1.5% and 4%) [2, 4, 13, 14]. The low rate of UTIs in our group may be due to two factors: our study is based on the ERP setting, which is reported to reduce medical postoperative morbidity [15]. Okrainec et al. [16] specifically assessed the occurrence of UTIs in the context of ERP, and they reported a UTI rate of 0.8%, which is similar to ours; for non‐compliance patients, they found that the UTIs occurred in 4.1% of patients. Another possibility is that our patients all had a UC placed for a short period of time. Short‐course management is associated with a reduction in UTIs [10] and this may therefore explain the low rate of UTIs in our cohort.

The number of patients with AUR in our cohort was at the lower limit of the rates reported in the literature. Our study design may have led to an underestimation, and this indicates that, as it is currently being carried out, it is feasible to place a UC for a short period of time or to not place one at all. The literature agrees with this conclusion—in a small cohort of colon or high rectum anastomosis patients, the authors concluded that not using UCs was feasible as they reported an acceptable AUR rate of 9.2% [3]. A recent cohort study reported that the amount of time in which the UC is in position, or the complete absence of a UC, were not risk factors for AUR [2]. We observed the same results, which showed that UC management was not a risk factor for AUR or UTIs.

Our results show no difference in the incidence of AUR if a UC is not placed compared to if a UC is placed for <24 h. As one of the aims of the urinary drainage is to prevent the occurrence of AUR [5], UC is therefore not mandatory if the physician usually proposes short‐course management of UC for these indications. As we included only patients with short‐course management of UC, we cannot report on the benefits of no UC placement or UC placement <24 h in comparison with UC placement >24 h.

Given that no UC placement is not associated with increased AUR, the absence of a UC could have some beneficial effect as it could improve the perioperative comfort of the patients. Also, the proper management of urinary drainage is associated with better compliance and optimal ERP results [17] and compliance with ERPs plays a key role in reducing the length of stay, postoperative morbidity and the occurrence of postoperative ileus [15, 18, 19].

As the absence of a UC was not significantly associated with a reduction in the incidence of UTI, our hypothesis was not confirmed. One of the objectives of no UC placement is to reduce the incidence of UTI because catheter‐associated UTI costs represent a financial burden which costs anywhere between $1000 to $10 000 per patient [20]. The benefit–risk ratio should therefore be discussed and weighted according to the situation (hour of the end of surgery, difficulty of the surgery etc.) as well as to the patients.

Some patients, such as older male patients with an ASA score ≥3, are at an increased risk for AUR [21]. Epidural analgesia has been reported as a risk factor for AUR [12], but it would have no place in an ERP context as it inhibits the recovery of colorectal patients [22]. Even in those situations, the management of UCs was not an independent risk factor for AUR and removing UCs altogether should probably be favoured.

This study does have some limitations inherent to its retrospective design. Certain pieces of information, such as the prevention of AUR management or the diagnosis of UTIs, that may not have been collected if their effects were judged too minimal by the audit reporter, are lacking. Some research has shown that the outcomes in colorectal surgery can depend on the very definition of UTIs [23]. Also, the timing of UC removal may not be similar in each centre, as some surgeons remove the UC immediately on the table while others remove it after 6, 12 or 24 h. Additionally, no information on the failure of cases in which no UC was in place were collected in terms of difficulty during surgery. In pelvic surgery, the discomfort caused by the bladder could lead to a UC being installed during the operation. Some UCs can be installed to collect urine samples in order to assess the fluid loading. There was no information on the timing of AUR. AUR can occur immediately after surgery and could be considered as primary but it could also occur postoperatively after a few days and be associated with postoperative ileus or intrapelvic complications [6]. Finally, some of the patients might have had emergency surgery, something that is not identified by the GRACE audit database.

Despite these biases, this study allows strong conclusions to be made on the feasibility of the early removal and abstention of UCs, and it provides some information in order to build randomized controlled studies. Further studies will have to determine if, compared to ≤24 h or >24 h UC, the abstention of UC placement improves the comfort of the patient or if it leads to increased unplanned UC placement during surgery or to increased problems during hospitalization.

## CONCLUSION

UCs in place for <24 h do not reduce the occurrence of AUR or UTI compared to the absence of UCs in the current practice in ERP settings. No placement of UC could be considered in young female patients with ASA score <2.

## ETHICAL APPROVAL

The database was approved by the French National Data Protection Authority (2014#1817711).

## AUTHOR CONTRIBUTIONS

Conception: AV, JFH, KS. Planning: JFH, AV, KS. Carrying out: AV, JFH, JG, EC, JMR, JJ, KS. Analysis: AV, JFH, JG. Writing up: AV, JFH, KS. Critically revising manuscript for important intellectual content: AV, JFH, JG, EC, JMR, JJ, KS. Final approval of the version to be published: AV, JFH, JG, EC, JMR, JJ, KS.

## CONFLICT OF INTEREST

Professor Venara declares a conflict of interest with Takeda, Coloplast, ThermoFisher, Biom'up, Sanofi‐Aventis (consulting and lecture). The other authors declare no conflict of interest.

## INFORMED CONSENT

Written notification and oral informed consent was collected from patients prior to the entry of their data in the database.

## Data Availability

The data that support the findings of this study are available from the corresponding author upon reasonable request.
